# Inflammatory protein profiling in immune cells identifies molecular signatures for enhanced diagnostic precision in psoriatic arthritis

**DOI:** 10.1186/s43556-026-00432-5

**Published:** 2026-03-19

**Authors:** Jesús Eduardo Martín-Salazar, Iván Arias-de la Rosa, María Dolores López-Montilla, Pedro Ortiz-Buitrago, Laura Cuesta-López, María Ángeles Puche-Larrubia, Miriam Ruiz-Ponce, Carlos Pérez-Sánchez, Antonio Manuel Barranco, Adrián Santiago Ortiz, María Carmen Ábalos-Aguilera, Laura Romero-Zurita, Rafaela Ortega, Elena Moreno-Caño, Jerusalem Calvo, Alejandro Escudero-Contreras, Chary López-Pedrera, Eduardo Collantes-Estévez, Clementina López-Medina, Nuria Barbarroja

**Affiliations:** 1 Rheumatology Service/Department of Medical and Surgical Sciences, Maimonides Institute for Research in Biomedicine of Cordoba (IMIBIC)//University of Cordoba/Reina Sofia University Hospital, Córdoba, Spain; 2 Department of Gastroenterology, Hospital General de Tomelloso, Tomelloso, Spain; 3 Instituto de Investigación Sanitaria de Castilla-La Mancha (IDISCAM), Toledo, Spain; 4 Cobiomic Bioscience S.L, Córdoba, Spain; 5 Department of Cell Biology, Physiology and Immunology, Maimonides Institute of Biomedical Research of Cordoba (IMIBIC), Reina Sofia University Hospital, University of Córdoba, Córdoba, Spain; 6 Bioinformatics and Biostatistics Unit, Instituto Maimónides de Investigación Biomédica de Córdoba (IMIBIC), Córdoba, Spain

**Keywords:** Proteomic, Machine Learning, Biomarkers, Psoriatic Arthritis

## Abstract

Psoriatic arthritis (PsA) lacks reliable biomarkers to support early diagnosis and disease stratification. This study aimed to identify protein signatures associated with PsA and develop a clinically relevant model integrating molecular and clinical features. We conducted a cross-sectional study including 143 patients with PsA and 101 controls across three cohorts. The exploratory cohort included 77 patients with PsA and 50 symptomatic controls without inflammatory disease. Two independent cohorts were used for external validation: validation cohort 1 comprised PsA patients receiving conventional DMARDs, and validation cohort 2 included PsA patients receiving biological treatment. Proteomic profiling of 384 inflammation-related proteins was performed in PBMCs using Olink technology. Machine learning–based approaches were applied to identify and optimize a diagnostic biomarker model integrating proteomic and clinical variables. Functional in vitro assays were conducted to evaluate the mechanistic regulation of biomarker-associated proteins. Sixty-eight proteins were significantly dysregulated in PsA, highlighting activation of immune-inflammatory pathways. Several proteins correlated with arthritis severity, psoriasis burden, and CRP. A five-parameter diagnostic model (ESR, plaque psoriasis, LILRB4, ADGRE2, SIGLEC10) showed excellent discrimination across all three cohorts. The upregulation of LILRB4, ADGRE2, and SIGLEC10 in PBMCs exposed to PsA serum supported their mechanistic relevance. Distinct proteomic signatures differentiated early from established PsA, revealing molecular patterns linked to disease progression. This study identifies a potential protein signature associated with PsA and provides a validated diagnostic model with high accuracy. These findings advance molecularly guided diagnosis and highlight biomarkers with both mechanistic and translational relevance.

**Supplementary Information:**

The online version contains supplementary material available at 10.1186/s43556-026-00432-5.

## Introduction

Psoriatic arthritis (PsA) is a chronic inflammatory musculoskeletal disease associated with psoriasis, presenting a wide range of clinical manifestations, both musculoskeletal and extra-musculoskeletal, and occurs in patients with either latent or manifest psoriasis. PsA is primarily characterized by its impact on the peripheral joints and entheses, although it can also involve the spine and sacroiliac joints. The average age of onset is around 40 years, and up to 5% of cases may present with mutilating arthritis, a severe, destructive, and deforming manifestation [1].

Despite the possible existence of a common pathogenic mechanism, patients with PsA exhibit a wide variety of clinical presentations (including the presence of dactylitis, enthesitis, severe psoriasis, and uveitis) as well as an uncertain course [2]. Patients may develop increased bone neoformation, leading to ankylosis and/or increased structural damage, or comorbidities that potentially put the patient's life at risk, particularly cardiovascular risk [3]. Just as there is significant clinical variability, a wide range of molecular pathways is involved in PsA [4].

Due to the diverse manifestations of PsA and the frequent presence of unspecific symptoms, especially in early stages of the disease, achieving an early diagnosis remains challenging [5]. At present, available detection markers primarily rely on clinical data. However, considering the variability of the pathways involved in the diseases, it seems logical to integrate clinical and molecular data. In this line, given the complexity and variability of PsA, the development of new molecular analysis tools, may contribute to the identification of new protein biomarkers associated with the disease, as well as enhance its biological understanding and treatment [6, 7]. However, only a few studies have explored or defined the molecular profiles associated with PsA [8]. In this context, a recent study conducted by our research group identified inflammation- and cardiovascular disease–related proteins potentially involved in disease pathogenesis [9].

Immune cells, particularly peripheral blood mononuclear cells (PBMCs), play a fundamental role in the inflammatory and immune-mediated mechanisms underlying PsA [10]. Advances in molecular profiling technologies combined with computational approaches now allow the identification of protein signatures and molecular subgroups that may relate to clinical manifestations. These strategies can improve patient stratification, support earlier recognition of the disease, and potentially highlight biomarkers or pathways relevant for diagnosis or therapeutic intervention [11].

Within this framework, Olink’s Proximity Extension Assay (PEA) has emerged as a robust platform for proteomic biomarker discovery. Its high sensitivity, specificity, and capacity to quantify numerous inflammation-related proteins from minimal sample volumes make it well suited for studying immune-mediated diseases such as PsA [12]. In parallel, artificial intelligence (AI) and machine learning (ML) methods have transformed biomedical research by enabling the integration and interpretation of complex datasets, improving the identification of diagnostic patterns, and enhancing predictive modelling [13].

In summary, the present study aims to: a) identify proteomic profiles or signatures in the PMBCs of PsA patients, linked to clinical characteristics of the disease; b) evaluate the proteomic profile of the patient cohort based on the duration of disease progression and c) to identify a biomarker combining clinical and molecular features through machine learning approaches serving as potential diagnostic tools, leveraging advanced protein analysis technologies. This strategy enhances clinical-molecular integration, paving the way for a more comprehensive and efficient approach to disease management.

## Results

### Unsupervised clustering reveals three molecularly distinct clinical phenotypes

**Fig. 1:**
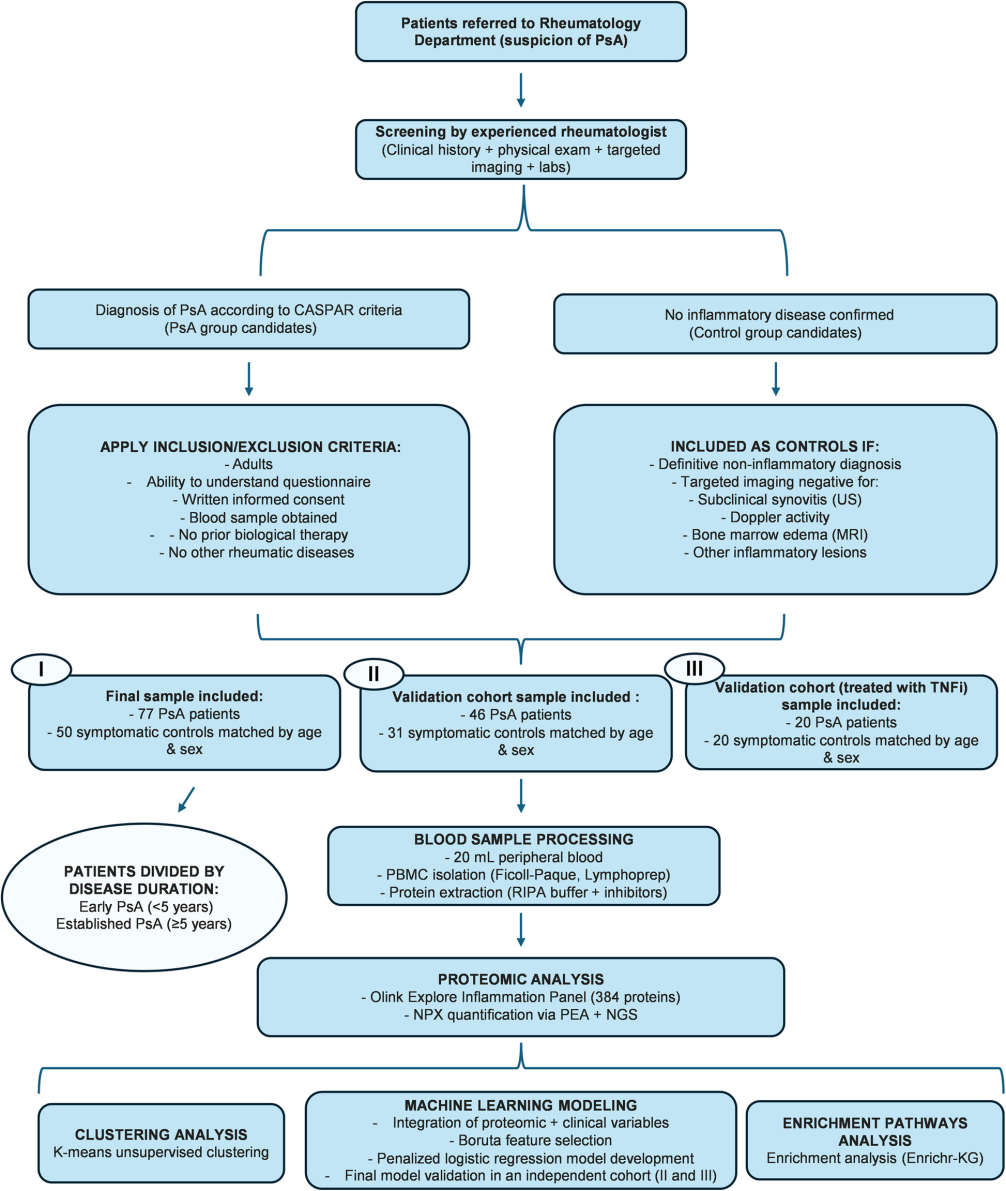
Overview of the study workflow. Flow chart summarizing patient selection, cohort assembly, proteomic analysis, machine-learning modeling

**Fig. 2:**
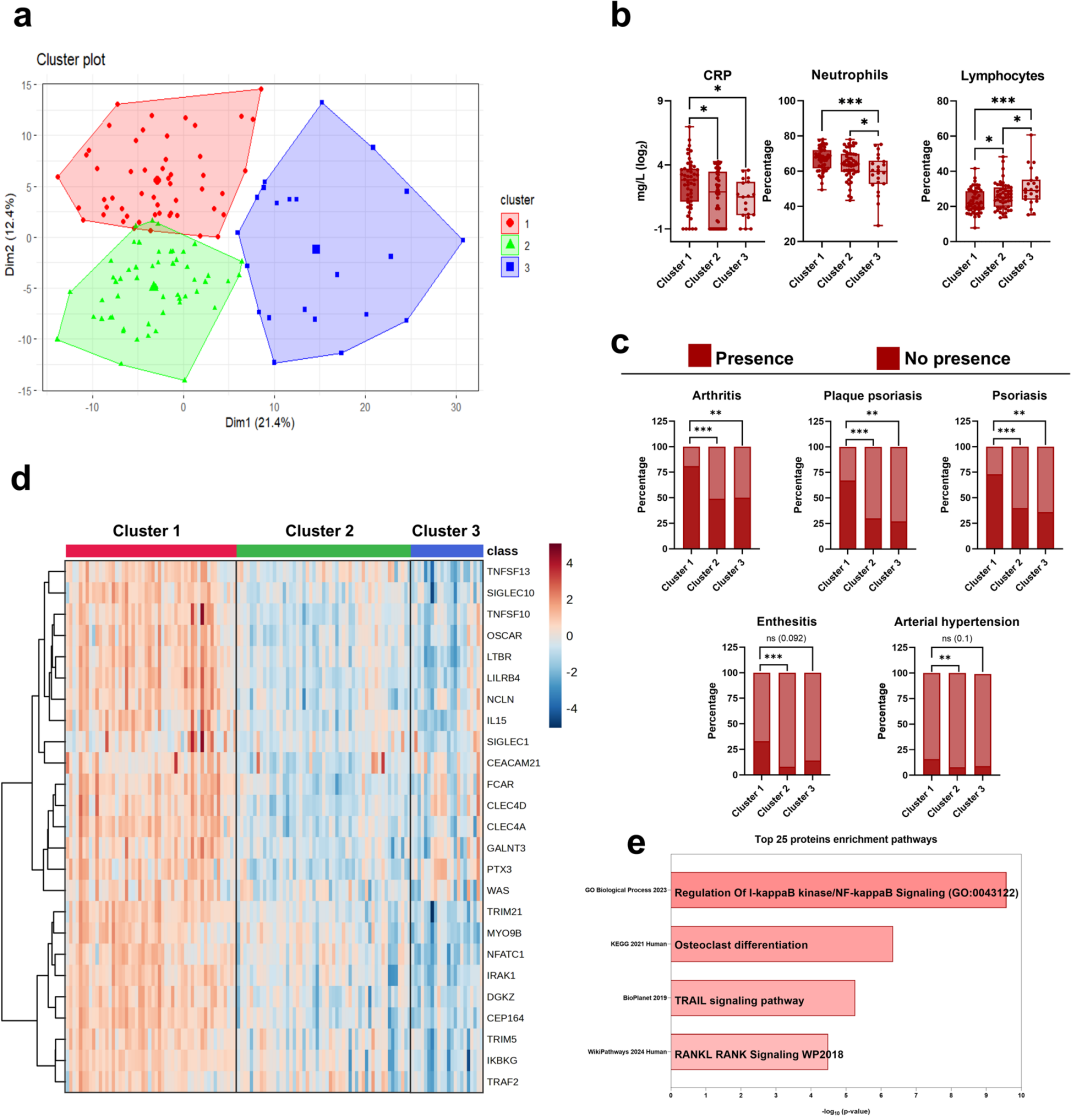
Unsupervised Proteomic Analysis Revealed Three Distinctive Molecular Cluster of Individuals in the entire cohort. **a** Cluster plot obtained using the k-means method. **b** Differences in CRP, neutrophil, and lymphocyte levels among the three clusters. **c** Differences in the proportion of arthritis, plaque psoriasis, psoriasis, enthesitis, and hypertension among the three clusters. **d** Heatmap of the top 25 proteins with the most significant expression differences between clusters. **e** Enrichment analysis of the top 25 proteins with the most significant expression differences between clusters. *p-value < 0.05; **p-value < 0.01; ***p-value < 0.001. Population size: Cluster 1 (n = 52); Cluster 2 (*n* = 53); Cluster 3 (*n* = 22)

An unsupervised clustering analysis was performed on the entire cohort, including both diagnosed patients and symptomatic controls (Fig. 1), to determine whether individuals could be stratified based on proteomic expression patterns derived from PBMCs isolated from PsA patients and symptomatic controls, independently of their clinical diagnosis. The analysis revealed three well-differentiated clusters (Fig. 2a). The clinical details of each cluster are presented in Table 1.

**Table 1:** Clinical and laboratory characteristics of the clusters

	**Cluster 1 (n = 52)**	**Cluster 2 (n = 53)**	**Cluster 3 (n = 22)**
Female/Male (n/n)	23/29	36/17	12/10
Age (years)	43.3 ± 10.9	41.9 ± 12.2	38.5 ± 12.1
Disease duration (years)	6.9 ± 5.5	5.7 ± 4.2	6.0 ± 4.5
Disease activity and inflammatory markers			
DAPSA	23.7 ± 12.5	23.8 ± 8.8	21.8 ± 13.3
BASDAI (0–10)	6.0 ± 1.9	5.6 ± 2.5	4.8 ± 1.9
CRP (mg/L)	5.7 ± 3.5^ab^	3.7 ± 3.1	3.8 ± 4.0
ESR (mm/1 h)	20.4 ± 18.6	11.1 ± 9.8	25.7 ± 34.4
Clinical manifestations/Comorbidities			
Psoriasis (%)	73.1^ab^	39.6	36.4
Plaque psoriasis (%)	67.3^ab^	30.2	27.3
Inverted psoriasis (%)	23.1	11.3	13.6
Nail psoriasis (%)	32.2	17.0	22.7
Palmoplantar psoriasis (%)	5.8	1.9	4.5
Scalp psoriasis (%)	46.2^ab^	18.9	22.7
Dactilytis (%)	38.5	24.5	31.8
Uveitis (%)	1.9	5.7	4.5
Enthesitis (%)	32.7^ab^	7.5	13.6
Arthritis (%)	80.8^ab^	49.1	50.0
IBD (%)	5.8	7.5	0.0
Arterial hypertension (%)	13.5^ab^	7.5	9.1
Obesity (%)	34.9	31.6	26.3
Diabetes (%)	5.8	7.5	9.1
Dyslipidemia (%)	7.4	7.5	4.5
Treatments			
NSAIDs (%)	78.8	60.4	68.2
Methotrexate (%)	44.2	22.6	18.2
Corticosteroids (%)	32.7	28.3	27.3
Sulfasalazine (%)	1.9	0	9.1

Clusters were defined using unsupervised k-means clustering based on protein expression levels measured in PBMCs isolated from PsA patients and symptomatic controls. Data are represented by mean ± SD. *DAPSA* Disease Activity Score for Psoriatic Arthritis, *BASDAI* Bath Ankylosing Spondylitis Disease Activity Index, *CRP* C-Reactive Protein, *ESR* Erythrocyte Sedimentation Rate, *IBD* Inflammatory Bowel Disease, *NSAIDs* Non-steroidal anti-inflammatory drugs

^a^Significant differences respect to Cluster 2

^b^Significant differences respect to Cluster 3

*p*-value < 0.05

Cluster 1 included 52 individuals (44 patients and 8 controls), cluster 2 consisted of 53 individuals (23 patients and 30 controls), and cluster 3 comprised 22 individuals (10 patients and 12 controls). Notably, the majority of patients were found in cluster 1, which also had the lowest presence of controls.

Clinically, cluster 1 exhibited significantly higher levels of C- reactive Protein (CRP), a higher percentage of neutrophils, and a lower percentage of lymphocytes (Fig. 2b). Additionally, of all the clinical manifestations and comorbidities analyzed, we observed that cluster 1 showed a higher occurrence of arthritis, psoriasis (specifically plaque psoriasis), enthesitis, and arterial hypertension (Fig. 2c).

Furthermore, proteins with the most significant differences in expression between cluster 1 and the other clusters were identified. The 25 most differentially expressed proteins were found to be upregulated in cluster 1 compared to the others (Fig. 2d). Enrichment analysis revealed alterations in pathways related to regulation of I-kappaB kinase/nuclear factor kappa B (NF-κB) signaling, osteoclast differentiation, TNF-related apoptosis-inducing ligand (TRAIL) signaling, and receptor activator of nuclear factor kappa B ligand (RANKL)–RANK signaling*,* indicating disruptions in inflammatory processes and bone destruction (Fig. 2e).

### Molecular links between psoriasis, arthritis, and systemic inflammation: shared and disease-specific patterns

**Fig. 3:**
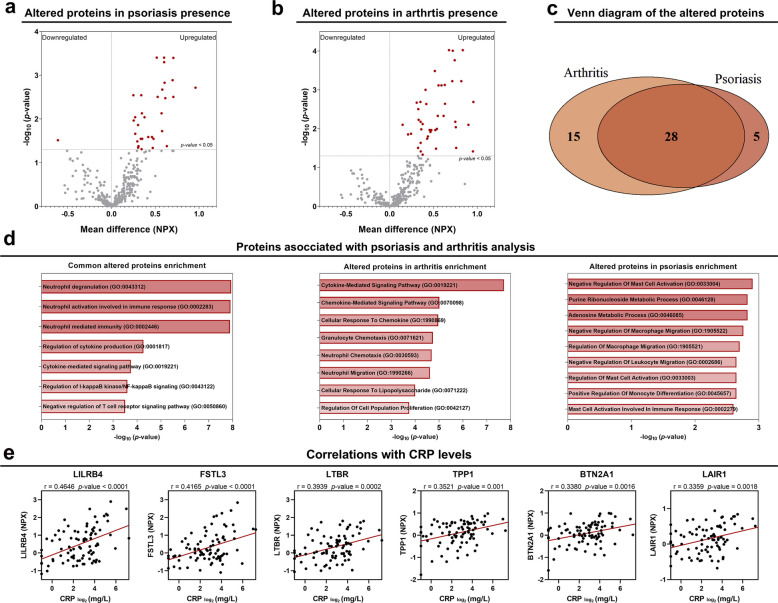
Association study of the proteomic profile with clinical characteristics. **a** Volcano plot of proteins significantly altered in the presence of psoriasis. **b** Volcano plot of proteins significantly altered in the presence of arthritis. **c** Venn diagram of proteins significantly altered in the presence of psoriasis and arthritis. **d** Enrichment analysis of proteins significantly altered in psoriasis, arthritis, and those commonly altered in both conditions. **e** Pearson correlations between CRP levels and protein expression levels

An association study was conducted on the entire cohort between the altered inflammatory proteome and the clinical characteristics of the disease, focusing on the relationship between the normalized expression of each protein and the presence of clinical features. This comparison identified 33 proteins with altered expression in individuals with psoriasis versus those without (Fig. 3a and Fig. S1a), and 43 in individuals presenting with arthritis compared to those without. (Fig. 3b and Fig. S1b). We identified which of these proteins were commonly altered in both manifestations and which were specifically altered in each condition. A total of 28 proteins were found to be altered in both manifestations, while 15 were specifically altered in presence of arthritis and 5 in presence of psoriasis (Fig. 3c). Enrichment analysis revealed dysregulation of cytokine signaling pathways associated with arthritis, alterations in mast cell activation in psoriasis, and disruptions in neutrophil-related pathways in both manifestations (Fig. 3d).

Additionally, bivariate correlations were performed to study the association between the previously highlighted proteins and the different quantitative clinical variables collected during the study. The results showed significant positive correlations between six proteins: leukocyte immunoglobulin-like receptor B4 (LILRB4), follistatin-like 3 (FSTL3), lymphotoxin beta receptor (LTBR), tripeptidyl peptidase 1 (TPP1), butyrophilin subfamily 2 member A1 (BTN2A1), and leukocyte-associated immunoglobulin-like receptor 1 (LAIR1), and the levels of CRP (Fig. 3e), suggesting an association between these proteins and the systemic inflammation.

### Altered proteomic profile of PBMCs in patients with PsA

Patients with PsA (39 women and 38 men, mean age 43.5 ± 9.3 years) had a mean disease duration of 6.4 ± 5 years since symptom onset, with a mean disease activity score of 22.7 ± 11.9 (moderate) according to the disease activity in psoriatic arthrtis (DAPSA) scale. No significant differences in age and sex were observed between PsA patients and the control cohort (32 women and 18 men, mean age 39.3 ± 14.2 years). PsA patients exhibited significantly elevated levels of acute-phase inflammatory markers, such as CRP and erythrocyte sedimentation rate (ESR), compared to controls. Additionally, there was a higher prevalence of psoriasis, dactylitis, enthesitis, arthritis, and arterial hypertension in PsA patients, as well as a greater use of specific treatments compared to controls (Table 2).

**Table 2:** Clinical and laboratory characteristics of the PsA patients and symptomatic controls

	**PsA patients (n = 77)**	**Symptomatic controls (n = 50)**
Female/Male (n/n)	39/38	32/18
Age (years)	43.5 ± 9.3	39.3 ± 14.2
Disease duration (years)	6.4 ± 5	-
Disease activity and inflammatory markers		
DAPSA	22.7 ± 11.9	-
BASDAI (0–10)	5.7 ± 2.0	-
CRP (mg/L)	14.2 ± 23.9*	4.1 ± 5.3
ESR (mm/h)	23.3 ± 23.7*	8.2 ± 7.7
Clinical manifestations/Comorbidities		
Psoriasis (%)	81.8*	8.0
Plaque psoriasis (%)	71.4*	4.0
Inverted psoriasis (%)	27.3*	0
Nail psoriasis (%)	39.0*	2.0
Palmoplantar psoriasis (%)	5.2	2.0
Scalp psoriasis (%)	49.4	2.0
Dactylitis (%)	50.6*	2.0
Uveitis (%)	1.3	8.0
Enthesitis (%)	29.9*	2.0
Arthritis (%)	97.4*	8.0
IBD (%)	3.9	8.0
Arterial hypertension (%)	11.7*	8.0
Previous low back pain (%)	19.5*	90
Obesity (%)	32.8	30.6
Diabetes (%)	7.8	6.0
Dyslipidemia (%)	9.1	4.0
Treatments		
NSAIDs (%)	83.1*	48
Methotrexate (%)	49.4*	2
Leflunomide (%)	16.9*	0
Corticosteroids (%)	45.5*	6
Sulfasalazine (%)	2.6	2

Data are represented by mean ± SD. *PsA* Psoriatic Arthritis, *DAPSA* Disease Activity Score for Psoriatic Arthritis, *BASDAI* Bath Ankylosing Spondylitis Disease Activity Index, *CRP* C-Reactive Protein, *ESR* Erythrocyte Sedimentation Rate, *IBD* Inflammatory Bowel Disease, *NSAIDs* Non-steroidal anti-inflammatory drugs

^*^Significant differences respect to controls, *p*-value < 0.05

**Fig. 4:**
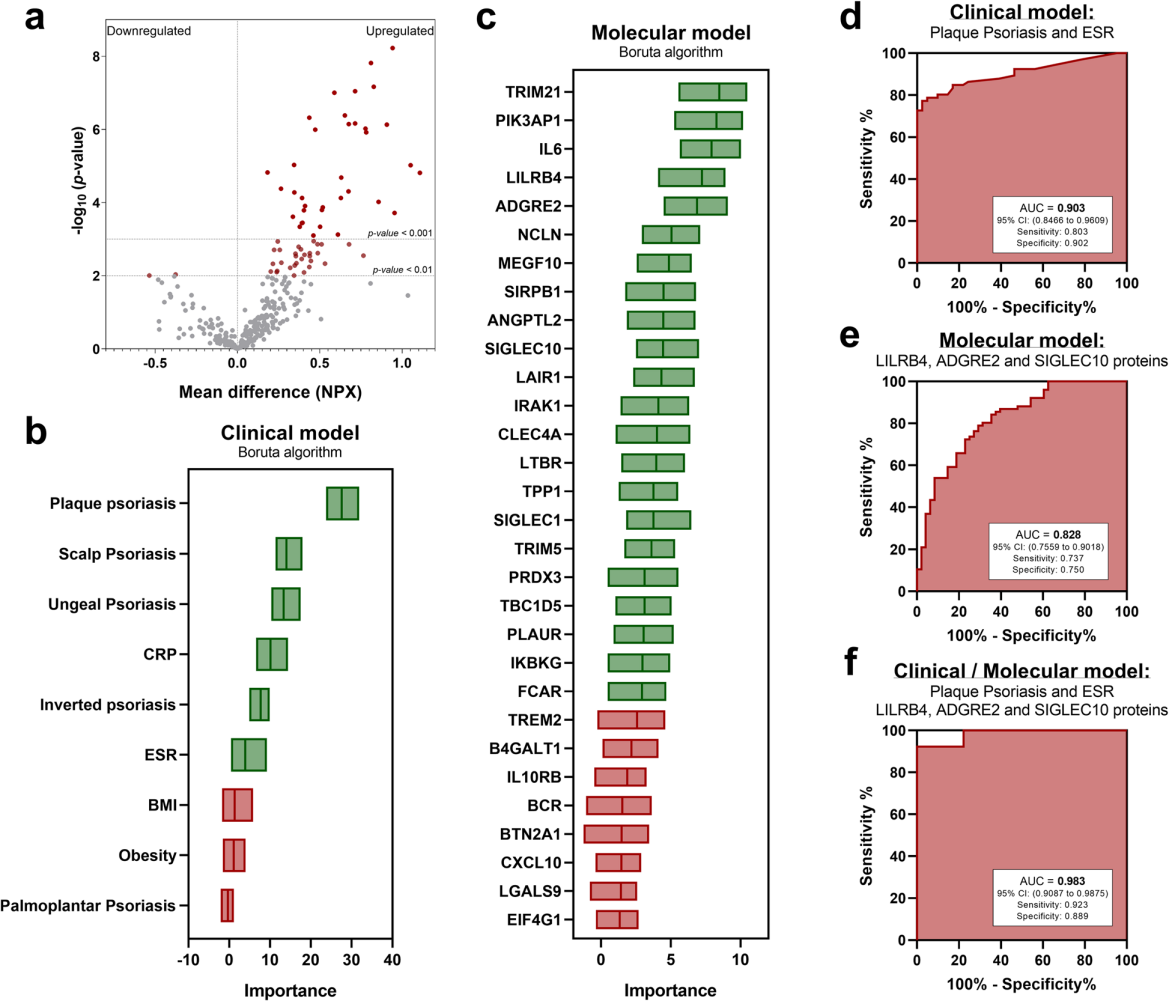
Machine learning predictive model integrating clinical and molecular variables. **a** Volcano plot of proteins significantly altered in PsA patients compared to symptomatic controls. **b** Importance of clinical variables identified using the Boruta algorithm. Green: Variable confirmed as important; Red: Variable confirmed as not important**. c** Importance of molecular variables identified using the Boruta algorithm. Green: Variable confirmed as important; Red: Variable confirmed as not important**. d** ROC curve of the model based on clinical variables. **e** ROC curve of the model based on molecular variables. **f** ROC curve of the model combining clinical and molecular variables

To analyze potential molecular patterns associated with PsA, an extensive proteomic analysis was conducted on PBMCs from PsA patients compared to a control group of individuals with musculoskeletal manifestations but without PsA. Out of a total of 384 proteins analyzed, 68 proteins were found to be significantly altered in PsA patients after adjustment by False Discovery Rate (FDR) (Fig. 4a). Of these, 66 were increased and 2 were decreased in PsA patients.

The altered proteins were ranked according to their statistical significance, with the 36 most significant proteins (p-value < 0.001) highlighted in Fig. S1c. The remaining significantly altered proteins can be found in Table S1.

### Machine learning integration of proteomic and clinical data for identifying diagnostic biomarkers in psoriatic arthritis

To assess whether the combination of the studied protein levels and clinical data could differentiate between PsA patients and controls (those with clinical manifestations but without rheumatic disease), a predictive model was developed integrating proteomic and clinical data. Using a machine learning approach, relevant features were selected through logistic regression, and a model was trained with 80% of the data, reserving the remaining 20% for testing and validation.

First, we identified the clinical (Fig. 4b) and molecular (Fig. 4c) variables that are most relevant for distinguishing patients from controls. Then, we aimed to identify clinical and molecular models that, on their own, had a strong ability to discriminate between patients and controls. The combination of clinical variables with the highest discriminatory power was plaque psoriasis and ESR levels, achieving a combined Area Under Curve (AUC) of 0.903 (Fig. 4d). At the molecular level, the combination of three proteins—LILRB4, adhesion G protein-coupled receptor E2 (ADGRE2), and sialic acid-binding immunoglobulin-like lectin 10 (SIGLEC10)—demonstrated good discriminatory ability, with an AUC of 0.828 (Fig. 4e).

Next, we developed a model that integrated both clinical and molecular variables. The final model included the three proteins (LILRB4, ADGRE2, SIGLEC10), ESR, and the presence of plaque psoriasis. This model demonstrated high discriminatory capacity with an AUC of 0.98 (Fig. 4f). Additionally, it showed a sensitivity of 92.31% and a specificity of 88.89%, indicating its potential utility as a diagnostic tool.

**Fig. 5:**
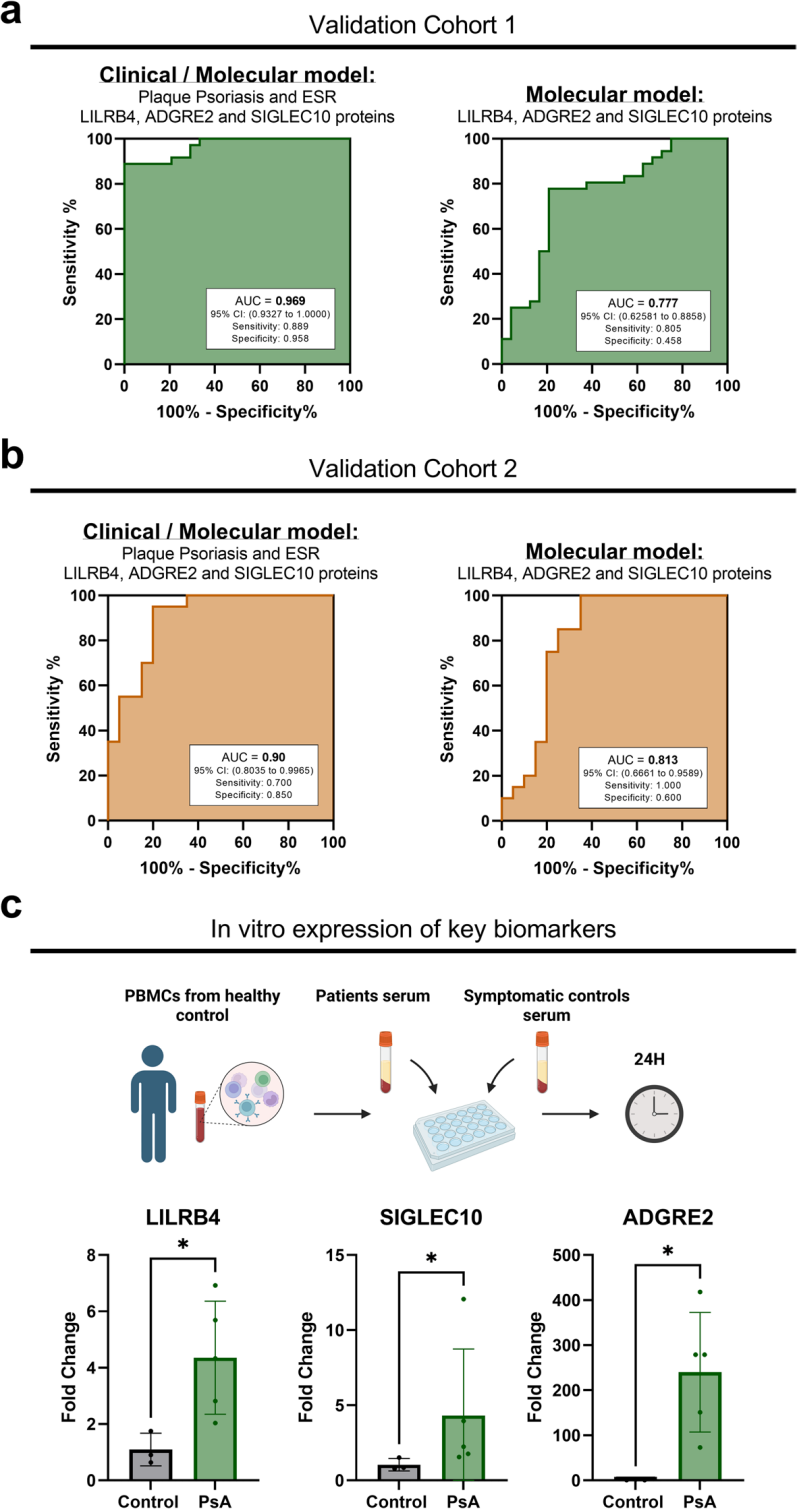
External validations and in vitro assessment of key biomarkers. **a** ROC curves for the clinical–molecular and molecular models in Validation Cohort 1**. b** ROC curves for the clinical–molecular and molecular models in Validation Cohort 2 (treated with TNFi). **c** In vitro fold-change expression of LILRB4, SIGLEC10, and ADGRE2 in PBMCs exposed to serum from PsA patients or symptomatic controls

To evaluate the generalizability of the predictive model, two independent external validation cohorts were analyzed (Fig. 1). The first validation cohort included 46 PsA patients and 31 symptomatic controls, matched by age and sex and evaluated following the same criteria as the discovery cohort. The clinical characteristics of this cohort are shown in Table S2. When applied without recalibration, the clinical–molecular model achieved an AUC of 0.969, confirming excellent discriminative performance. The molecular model alone reached an AUC of 0.777, indicating that, even in a cohort with a high prevalence of psoriasis, the protein signature retains meaningful diagnostic value and contributes substantially to the strength of the combined model (Fig. 5a).

To further assess model robustness, we applied it to a second external cohort of 20 PsA patients (all receiving tumor necrosis factor inhibitors (TNFi) therapy) and 20 symptomatic controls, matched by age and sex. In this dataset, the clinical model achieved an AUC of 0.90, while the molecular model reached an AUC of 0.813 (Fig. 5b), both demonstrating good discriminative capacity beyond the discovery cohort. The clinical features of this cohort are presented in Table S3. This external validation supports the robustness of the predictive model, including its molecular component, across distinct clinical and therapeutic contexts.

### Serum-stimulated PBMC assay confirms upregulation of model proteins

For the in vitro validation assay (Fig. S2), serum samples from PsA patients and symptomatic controls were matched by age and sex to minimize potential confounding. The PsA group had a mean age of 43.90 ± 6,74 years and consisted of 3 women and 2 men, while the control group had a mean age of 44.22 ± 3.97 years and included 2 women and 1 man. The PsA patients selected for this experiment exhibited marked disease activity at the time of serum collection, with a mean CRP level of 78.42 ± 59.16 mg/L and a mean DAPSA score of 29.48 ± 9.43, ensuring that the sera accurately represented an active inflammatory environment.

PBMCs stimulated with serum from active PsA patients showed a consistent upregulation of the transcripts corresponding to the proteins included in the predictive model. In particular, *LILRB4*, *SIGLEC10*, and *ADGRE2* exhibited markedly increased expression in PBMCs exposed to PsA serum compared with those treated with serum from symptomatic controls (Fig. 5c). This transcriptional upregulation mirrors the proteomic differences identified in our patient cohort, strengthening the biological plausibility of these proteins as relevant markers within PsA-related inflammatory pathways.

### Molecular differences based on disease duration in PsA

To analyze whether there were differences in the proteomic profile of PBMCs in PsA patients based on disease duration, the 77 patients in the PsA cohort were divided into two groups based on the duration of the disease. One group, termed the early disease group (*n* = 34 patients), included those with less than 5 years of disease duration since the onset of the first musculoskeletal symptoms. The other group, defined as the established disease group (*n* = 43 patients), comprised patients with more than 5 years of disease duration since first symptoms appearance.

**Fig. 6:**
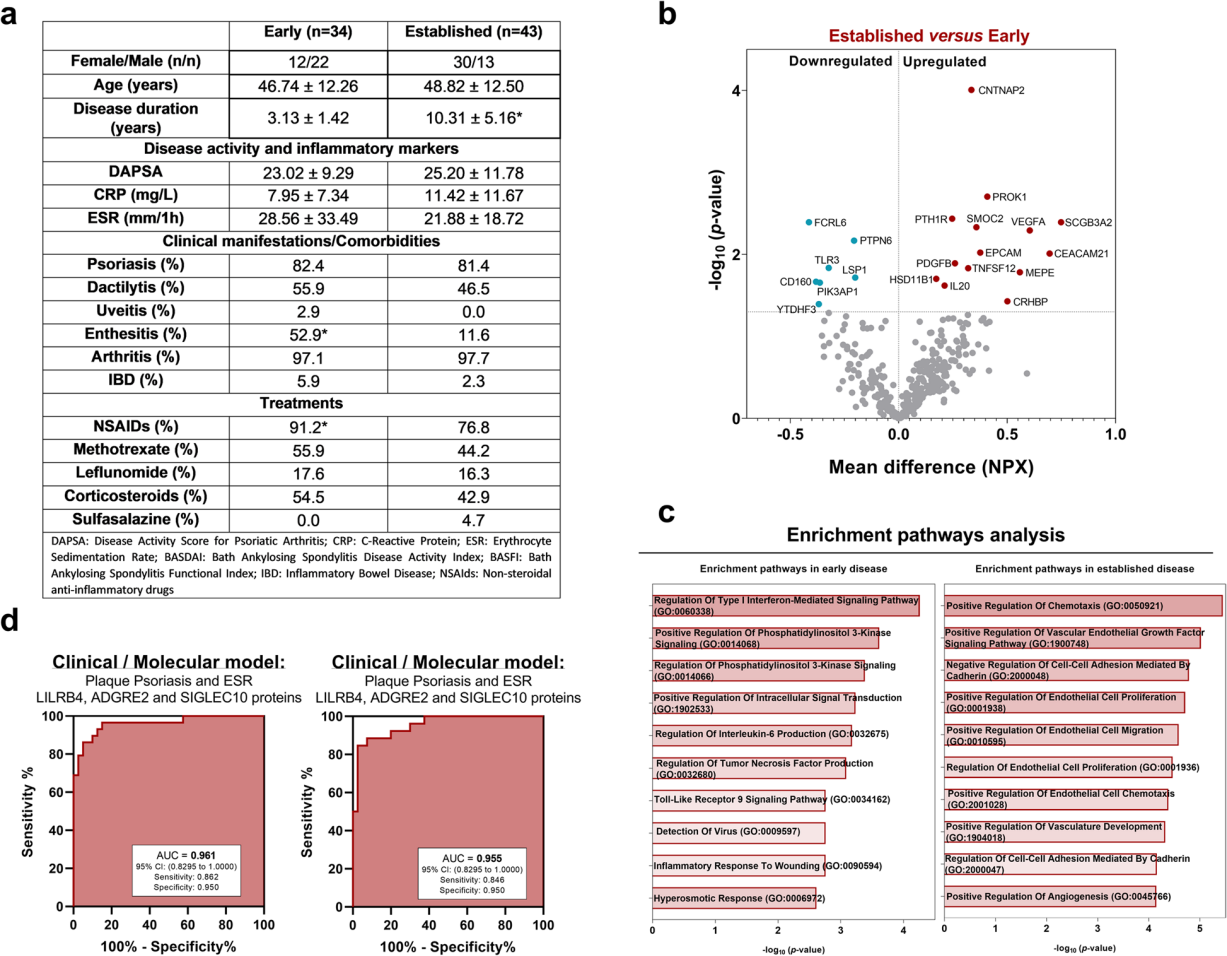
Application of a machine learning model to molecular profiles stratified by disease duration. **a** Descriptive table of the patient cohort, divided according to disease duration. **b** Volcano plot of proteins significantly altered when comparing patients with established disease to those with early disease. **c** Enrichment analysis of proteins altered in patients with established disease compared to those with early disease. **d** ROC curves of the model integrating clinical and molecular data, applied to the early and established disease cohorts

Comparison between the two groups revealed similar average ages, and both the early and established disease groups had moderate disease activity (23.02 ± 9.29 and 25.20 ± 11.78 points, respectively) according to the DAPSA scale. Of all the clinical variables and comorbidities analyzed, we observed that patients with established disease showed a lower prevalence of enthesitis compared to the early disease group. Regarding specific treatments, there were significant differences between the early and established disease groups. Patients with early disease had a higher average use of nonsteroidal anti-inflammatory drugs (NSAIDs) compared to the early disease group (Fig. 6a).

At the molecular level, we compared protein expression levels between the two groups to determine whether differences in the proteomic profile were associated with disease duration. A total of 21 altered proteins were identified when comparing patients with established disease to those with early disease, with 14 upregulated and 7 downregulated (Fig. 6b).

Enrichment analysis revealed alterations in pathways related to inflammatory processes in early disease patients, including *Regulation of Type I Interferon-Mediated Signaling Pathway, Regulation of Interleukin-6 Production,* and *Regulation of Tumor Necrosis Factor Production.* In contrast, patients with established disease showed enrichment in pathways associated with vascular processes, such as *Positive Regulation of Vascular Endothelial Growth Factor (VEGF) Signaling Pathway, Negative Regulation of Cell–Cell Adhesion Mediated by Cadherin, Positive Regulation of Endothelial Cell Proliferation,* and *Positive Regulation of Endothelial Cell Migration* (Fig. 6c)*.*

Finally, we evaluated whether the previously described diagnostic model maintained its efficiency when patients were stratified by disease duration, given the particular interest in accurately identifying patients in the early stages of the disease. The model achieved an AUC of 0.961 for early disease patients and 0.955 for those with established disease (Fig. 6d), confirming its potential as a reliable diagnostic tool in patients with early disease.

## Discussion

This study is, to our knowledge, the first to apply inflammatory proteomic profiling to PBMCs from patients with PsA and symptomatic controls without inflammatory disease. The analysis of 384 inflammation-related proteins revealed clear molecular differences that may contribute to improved diagnostic assessment. Although exploratory, these findings offer new insights into the proteomic landscape of PsA and suggest potential directions for future translational work.

In an initial unbiased analysis, we identified three molecular clusters within the full cohort. One cluster showed a higher burden of clinical manifestations, including arthritis and plaque psoriasis, as well as increased CRP levels. The proteins distinguishing this subgroup were enriched in inflammatory and tissue-related pathways, supporting previously described mechanisms in PsA and illustrating the relevance of molecular heterogeneity when evaluating patients with musculoskeletal symptoms.

Protein–phenotype association analyses further highlighted distinct signatures linked to arthritis and psoriasis, along with a shared neutrophil-related component. Proteins associated with arthritis were enriched in cytokine and chemokine pathways, consistent with literature in inflammatory arthritides [14–16]. Psoriasis-associated proteins were enriched in mast-cell pathways, in line with previous work implicating mast cells in disease initiation and progression [17, 18]. Shared proteins were linked to neutrophil-driven pathways, supporting earlier findings on neutrophil involvement in both psoriasis and PsA [19, 20]. Collectively, these observations emphasize the biological diversity underlying PsA phenotypes.

Since one of the main challenges in PsA diagnosis is the correct identification of patients in the early stages of the disease, it is important to explore how clinical and molecular characteristics differ according to disease duration. Previous studies have mainly characterized clinical differences between early and established PsA [21, 22]. Our study contributes to filling this gap by providing, proteomic profiles comparing patients with short disease duration and those with longer-standing PsA.

Proteins altered in early PsA were enriched in inflammatory pathways, consistent with the inflammation-driven mechanisms described in early disease stages [23]. In contrast, established PsA showed enrichment of vascular-related pathways, aligning with the recognized role of VEGF in PsA and with the increased cardiovascular risk documented in long-standing disease [24, 25]. These differences suggest that early disease may be characterized by higher inflammatory activity, while vascular and endothelial alterations may become more prominent over time.

We developed a diagnostic model for PsA by integrating clinical variables (ESR and plaque psoriasis) with three protein biomarkers (LILRB4, ADGRE2 and SIGLEC10) across three independent cohorts. The model was first generated in an exploratory discovery set and subsequently tested in two external validation cohorts, the second of which included patients receiving TNF inhibitors. Importantly, all cohorts used symptomatic controls rather than healthy individuals**,** reflecting the real-world population in which PsA screening occurs and providing a more clinically meaningful benchmark for diagnostic performance. This design allowed us to evaluate the robustness and generalizability of the molecular signature across distinct clinical situations, including patients under biologic therapy.

As expected, the combined clinical–molecular model achieved the highest AUC among all models evaluated. The inclusion of plaque psoriasis—previously reported as highly heterogeneous in severity and distribution [26] and one of the CASPAR criterion [27]—might raise concerns about potential circularity. However, psoriasis alone does not define PsA, as CASPAR classification requires fulfilling at least two additional criteria. More importantly, the clinical scenario in which such a diagnostic tool would be most impactful is precisely patients with psoriasis who present with musculoskeletal symptoms, where early PsA diagnosis remains a major unmet need. In this context, incorporating psoriasis might increase real-world applicability and reflect the target population where diagnostic uncertainty is greatest [28, 29].

Crucially, our findings also demonstrate that a purely molecular model composed exclusively of the three protein biomarkers retains high discriminative performance across all cohorts. The molecular-only model is particularly relevant for patients who do not present with psoriasis at diagnosis—up to 10–15% in some cohorts [30, 31]—and it helps mitigate concerns regarding dependency on clinical features in the combined model.

It is important to clarify that our model is diagnostic, not predictive, as it cannot determine whether these biomarkers anticipate future PsA in patients with psoriasis. Although a predictive model would have substantial clinical value, identifying high-risk individuals before joint damage occurs, such development requires prospective longitudinal cohorts, as shown in recent studies of preclinical immunological and clinical predictors of PsA [32, 33].

In addition, the observation that LILRB4, ADGRE2 and SIGLEC10 are upregulated in PBMCs upon exposure to PsA serum provides mechanistic support for their involvement in PsA-associated immune pathways, which aligns with prior functional studies linking these molecules to myeloid activation and regulation of inflammatory signaling [34, 35].

Despite these strengths, this study has several limitations. The sample size was modest, particularly in the early-disease subgroup, and larger multicenter cohorts will be required to confirm these findings. Additionally, PBMC-based proteomics, while informative, is not immediately applicable in clinical practice; future evaluation of the biomarker signature in serum and with more accessible platforms will be essential for translation. Although validation in two external cohorts supports the robustness of the model, further studies in more diverse populations are needed before considering clinical implementation. Moreover, we explicitly acknowledge that additional mechanistic and functional studies will be required to elucidate the biological roles of the identified proteins and to determine whether they may ultimately have therapeutic relevance.

In conclusion, this study expands the current understanding of PsA molecular features and proposes a combined clinical–molecular model that may assist in the diagnostic assessment of PsA, particularly in early or clinically uncertain cases. Continued validation and refinement will be necessary to determine its applicability in broader clinical settings.

## Methods

### Design and patients

An observational and cross-sectional study was conducted in a cohort of 77 patients diagnosed with PsA according to CASPAR criteria, consecutively recruited from the Rheumatology Department of Reina Sofía Hospital (Córdoba, Spain). A control group of 50 subjects with musculoskeletal symptoms (back pain, mechanical arthralgia or soft tissue pain), referred for suspected PsA or spondyloarthritis but with a definitive negative diagnosis, was also included.

To ensure that symptomatic controls truly lacked inflammatory disease, all individuals underwent targeted imaging according to their presenting symptoms. In patients referred for peripheral arthralgia, musculoskeletal ultrasound was performed to exclude subclinical synovitis or Doppler signal indicative of active inflammation. In those referred for low back pain, magnetic resonance imaging (MRI) of the sacroiliac joints and lumbar spine was conducted to rule out bone marrow edema or other inflammatory features. All patients were assessed by an experienced rheumatologist who confirmed or rejected the diagnosis of PsA based on clinical history, physical examination, imaging findings, and laboratory results. Importantly, if any diagnostic uncertainty persisted, the patient was not included as a control. Through this rigorous process, control subjects were confirmed to be free of objective inflammatory pathology. Control subjects were matched to PsA patients by age and sex.

Two independent external validation cohorts were additionally recruited to evaluate the generalizability of the predictive model. The first validation cohort included 46 PsA patients receiving conventional disease-modifying anti-rheumatic drugs** (**DMARDs) and 31 symptomatic controls, consecutively enrolled using the same clinical inclusion and exclusion criteria applied to the discovery cohort (including symptomatic controls). The second validation cohort consisted of 20 PsA patients under TNF-inhibitor treatment and 20 symptomatic controls, recruited following the same diagnostic workflow. Including this second cohort allowed the assessment of model performance in treated patients with biologics, ensuring validation across different therapeutic contexts (Fig. 1).

Patients and controls in both validation cohorts were matched by age and sex to minimize confounding effects. Serum samples and clinical data were collected following the same standardized procedures used in the primary cohort. The final predictive model derived from the discovery dataset was applied to each validation cohort without recalibration.

For the analysis based on disease duration, the cohort was divided into two groups: one with early disease and one with established disease. The cutoff was set at the median disease duration within the cohort, which was 5 years.

All included patients were adults, and individuals with other rheumatic diseases were excluded from both groups. Additionally, all recruited subjects provided written informed consent, which was specifically approved by the Ethics Committee of the Reina Sofía University Hospital (Reference Number 5694).

### Collected variables

During recruitment, sociodemographic data were collected. Previous treatments (NSAIDs, Corticosteroids, Sulfasalazine, Leflunomide), patient-reported questionnaire results the Bath Ankylosing Spondylitis Disease Activity Index (BASDAI), and the DAPSA score, were also recorded. The presence of clinical manifestations such as psoriasis, arthritis, dactylitis, enthesitis, uveitis, and previous low back pain was noted at the time of the visit, as well as the presence of comorbidities such as obesity, inflammatory bowel disease (IBD), dyslipidemia, arterial hypertension, and type II diabetes mellitus. In the evaluation of psoriasis, confirmed by a dermatologist, different subtypes were distinguished, including palmoplantar psoriasis, plaque psoriasis, inverse psoriasis, nail psoriasis, and scalp psoriasis. Blood tests were performed to measure CRP levels, ESR, and a complete biochemical profile.

### Blood sample collection, cell isolation, and protein extraction

At study initiation, a 20 mL peripheral venous blood sample was collected from each patient into tubes containing 3.2% sodium citrate as anticoagulant. PBMCs were isolated by density gradient centrifugation using Ficoll-Paque PLUS (density 1.007 g/mL) and Lymphoprep™ (Stemcell Technologies, Cambridge, UK), following the manufacturer’s instructions.

Proteins were extracted from the isolated PBMCs using a cold lysis protocol with Thermo Scientific™ RIPA buffer, supplemented with 15% protease inhibitors and 5% phenylmethylsulfonyl fluoride.

The cell suspension was incubated on ice for 20 min, with vortexing every 5 min. It was then centrifuged at 13,000 rpm for 10 min at 4 °C. The supernatant, containing the soluble proteins, was collected, quantified through Bradford method, and stored at −80 °C for further analysis.

### Proteomic analysis

The molecular characterization of PBMCs was performed using proteomic analysis through a Proximity Extension Assay (PEA) immunoassay provided by Olink Proteomics in Uppsala, Sweden. The protein concentration used was 0.5 µg/mL. Specifically, the Olink Explore Inflammation Panel was used, which allowed for the simultaneous measurement of 384 inflammation-related proteins in each sample. The results were expressed as Normalized Protein Expression (NPX) levels.

PEA is a dual-recognition immunoassay that employs paired antibodies, each conjugated to unique DNA oligonucleotides, to simultaneously bind a target protein in solution. Upon binding, the oligonucleotides hybridize and serve as a template for a polymerase-dependent DNA extension, generating a unique DNA sequence specific to the target protein. Protein quantification is then performed using next-generation sequencing (NGS). This method enhances the precision and sensitivity of protein detection in biological samples [36].

### Statistical and bioinformatic analysis

Unsupervised clustering was conducted based on the expression levels of all analyzed proteins using the k-means method, with the optimal number of clusters determined via the elbow method, employing the *nbclust* and *ggplot2* packages in R (v4.3.2). Group comparisons between PsA patients and controls were performed using Student’s t-test adjusted by FDR, following confirmation of data normality. Statistical significance was set at *p* < 0.01 (99% confidence). Pearson’s correlation coefficients were used to assess associations, considering *p* < 0.05 as significant. Analyses were performed using IBM SPSS Statistics 26.0 and GraphPad Prism 9.0.1.

Enrichment analysis was carried out using proteins with *p* < 0.001 via Enrichr-KG and Enrichr (Icahn School of Medicine at Mount Sinai). Proteomic data were integrated with clinical variables using an unsupervised machine learning approach in R, excluding arthritis and dactylitis due to their high diagnostic specificity. The objective was to identify biomarkers for early disease stages.

A predictive model was developed using Boruta feature selection and penalized logistic regression. First, Boruta was applied to the full set of protein variables to identify features classified as “important” based on Z-score comparisons with their shadow features. Only the confirmed important proteins were included in a logistic regression model, which was subsequently refined through stepwise backward elimination. The same Boruta-based procedure was then applied to the clinical variables, and the resulting selections were further reviewed by clinical experts to ensure medical plausibility; only variables meeting both Boruta confirmation and expert justification were retained.

Model training was performed using penalized logistic regression (glmnet) within the caret framework. Hyperparameter optimization of the alpha (regularization type) and lambda (regularization strength) parameters was conducted via repeated tenfold cross-validation (3 repeats). To avoid overfitting and obtain an unbiased estimate of model performance, 20% of the dataset was reserved as an independent test set and was not used during feature selection, training, or tuning. The final model was selected based on accuracy, specificity, and sensitivity, and its performance on the independent hold-out set was evaluated using AUC, sensitivity, and specificity.

### Functional in vitro validation of PsA-associated biomarkers

To experimentally validate the relevance of the key markers identified in the predictive model, an in vitro assay was performed using PBMCs (Fig. S2). PBMCs were isolated from a single healthy donor by density-gradient centrifugation and cultured under standard conditions. To reproduce the biological environment associated with PsA, PBMCs were stimulated with serum from PsA patients (*n* = 5) and symptomatic controls (*n* = 3).

Serum samples were selected to match patients and controls by age and sex. Only clinically active PsA patients were included to ensure that the experimental conditions accurately reflected the inflammatory milieu characteristic of the disease. PBMCs were incubated with 10% serum from each donor for 24 h. After stimulation, total RNA was extracted and reverse-transcribed, and gene expression levels of the selected biomarkers were quantified using quantitative PCR (qPCR). Expression levels were normalized to housekeeping genes and analyzed to determine whether the identified markers exhibited differential regulation under PsA-like conditions.

## Supplementary Information

Supplementary Material 1.

## Data Availability

The datasets used and/or analysed during the current study are available from the corresponding author on reasonable request.
