# Supplementary material for: Inflammatory protein profiling in immune cells identifies molecular signatures for enhanced diagnostic precision in psoriatic arthritis
Source: Mol Biomed. 2026 Mar 19;7:34. doi: 10.1186/s43556-026-00432-5 (PMC13000094; doi:10.1186/s43556-026-00432-5)
Supplement: Supplementary file 1 — Supplementary Material 1. [file 43556_2026_432_MOESM1_ESM.docx]

**Inflammatory protein profiling in immune cells identifies molecular signatures for enhanced diagnostic precision in Psoriatic arthritis.**

Jesús Eduardo Martin-Salazar, BSc^1^, Iván Arias-de la Rosa, PhD^1,2^, María Dolores López-Montilla, PhD^1^, Pedro Ortiz-Buitrago, BSc^1^, Laura Cuesta-López, BSc^1^, María Ángeles Puche-Larrubia, PhD^1^, Miriam Ruiz-Ponce, BSc^1^, Carlos Pérez-Sánchez, PhD^1,3,4^, Antonio Manuel Barranco, BSc^1^, Adrián Santiago Ortiz, BSc^5^, María Carmen Ábalos-Aguilera^1^, Laura Romero-Zurita, BSc^1^, Rafaela Ortega, PhD^1^, Elena Moreno-Caño, BSc^1^, Jerusalem Calvo, PhD^1^, Alejandro Escudero-Contreras, PhD^1^, Chary López-Pedrera, PhD^1^, Eduardo Collantes-Estévez, PhD^1^, Clementina López-Medina, PhD^1#^ and Nuria Barbarroja, PhD^1,3#*^.

^1^Rheumatology service/Department of Medical and Surgical Sciences, Maimonides Institute for Research in Biomedicine of Cordoba (IMIBIC)/ /University of Cordoba/ Reina Sofia University Hospital, Córdoba, Spain

^2^Department of Gastroenterology, Hospital General de Tomelloso, Tomelloso; Instituto de Investigación Sanitaria de Castilla-La Mancha (IDISCAM), Toledo, Spain

^3^Cobiomic Bioscience S.L, Córdoba, Spain

^4^Department of Cell Biology, Physiology and Immunology, Maimonides Institute of Biomedical Research of Cordoba (IMIBIC), Reina Sofia University Hospital, University of Córdoba

^5^Bioinformatics and Biostatistics Unit, Instituto Maimónides de Investigación Biomédica de Córdoba (IMIBIC), Córdoba, Spain

^#^These authors have contributed equally to this work.

^*^Corresponding author: Nuria Barbarroja Email: barbarrojan@gmail.com

**Supplementary information**

**Fig. S1 Proteins significantly altered in the group comparisons performed.**

**Fig. S2. In vitro experimental design.**

**Table S1. Proteins significantly dysregulated in PsA compared with symptomatic controls (p < 0.001)**

**Table S2. Clinical details of validation cohort 1**

**Table S3. Clinical details of validation cohort 2**

**
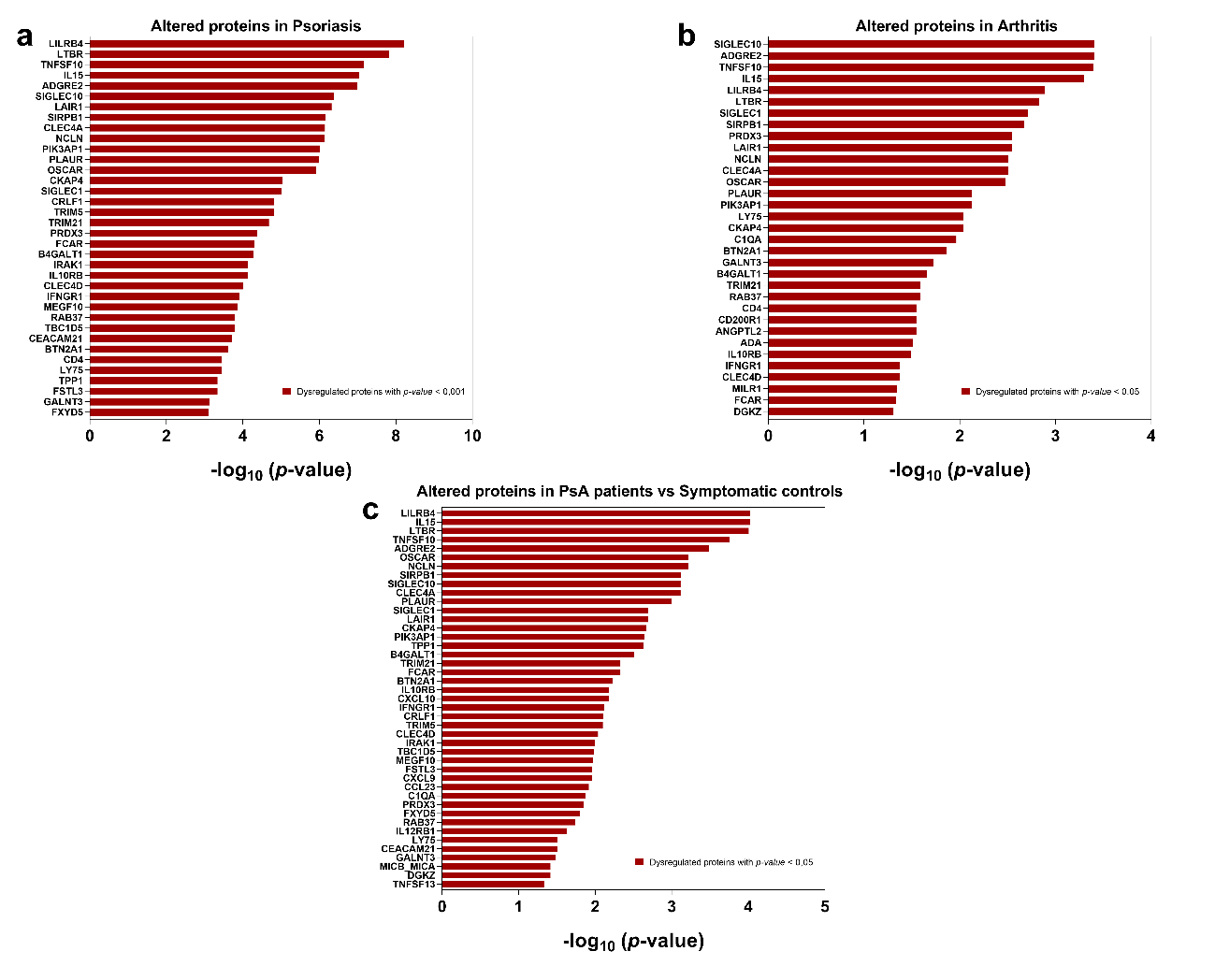
**

**Fig. S1.** **Proteins significantly altered in the group comparisons performed.** **(a)** Proteins significantly altered (*p*-value < 0.05) in the presence of psoriasis. **(b)** Proteins significantly altered (*p*-value < 0.05) in the presence of arthritis. **(c)** Proteins significantly altered (*p*-value < 0.001) in PsA patients compared to controls.

***
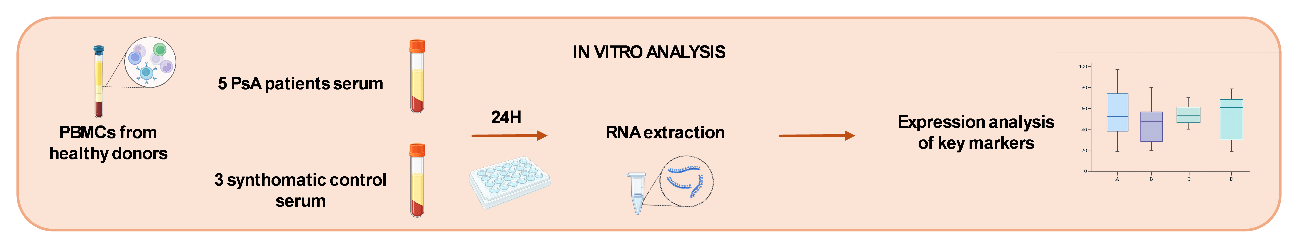
***

**Fig. S2. In vitro experimental design.** PBMCs isolated from healthy donors were exposed to serum from PsA patients to evaluate the regulation of candidate biomarker proteins. Subsequent analyses assessed changes in the expression of selected proteins associated with PsA

**Table S1. Proteins significantly dysregulated in PsA compared with symptomatic controls (p < 0.001)**

| **Proteins** | ***p*-value** |
| --- | --- |
| **CCL23** | 0.00113449 |
| **C1QA** | 0.00115398 |
| **BCR** | 0.00137848 |
| **CXCL10** | 0.00138102 |
| **ANGPTL2** | 0.00138325 |
| **TNFRSF4** | 0.00162405 |
| **DGKZ** | 0.00189977 |
| **ARNT** | 0.0019602 |
| **LGALS9** | 0.0019697 |
| **IKBKG** | 0.00241546 |
| **CEP164** | 0.00241713 |
| **CD200R1** | 0.00249402 |
| **TNFSF13** | 0.00267424 |
| **PTX3** | 0.00282618 |
| **PREB** | 0.00290135 |
| **PRKAB1** | 0.00290737 |
| **PRKCQ** | 0.00348819 |
| **NFATC1** | 0.00394821 |
| **ITM2A** | 0.00438733 |
| **CD79B** | 0.00455407 |
| **TRAF2** | 0.00467627 |
| **TNFRSF14** | 0.00472172 |
| **ICAM4** | 0.00525775 |
| **CXCL9** | 0.0057491 |
| **IL12RB1** | 0.00605899 |
| **LTO1** | 0.00741993 |
| **ANXA11** | 0.0076542 |
| **CD58** | 0.00795793 |
| **IL32** | 0.00813392 |
| **VASH1** | 0.00923634 |
| **ADA** | 0.00984464 |
| **CD70** | 0.00985372 |

**Table S2. Clinical details of validation cohort 1**

|  | **PsA (n=46)** | **Controls (n=31)** |  |
| --- | --- | --- | --- |
| **Female/Male (n/n)** | 26/20 | 18/13 |  |
| **Age (years)** | 46.42 ± 11.36 | 41.35 ± 14.35 |  |
| **Disease duration (years)** | 8.00 ± 5.16 | - |  |
| **Disease activity and inflammatory markers** | | | |
| **DAPSA** | 26.12 ± 12.36 | - |  |
| **CRP (mg/L)** | 13.56 ± 17.58* | 1.96 ± 5.21 |  |
| **ESR (mm/1h)** | 14.76 ± 13.26* | 7.57 ± 6.62 |  |
| **Previous lower back pain (%)** | 15.2* | 93.5 |  |
| **Clinical manifestations/Comorbidities** | | | |
| **Psoriasis (%)** | 95.6* | 6.5 |  |
| **Dactilytis (%)** | 40.0* | 0.0 |  |
| **Uveitis (%)** | 0.0 | 3.2 |  |
| **Enthesitis (%)** | 13.3* | 0.0 |  |
| **Arthritis (%)** | 79.1* | 6.5 |  |
| **IBD (%)** | 2.2* | 22.6 |  |
| **Arterial hypertension (%)** | 26.7 | 9.7 |  |
| **Treatments** | | | |
| **NSAIDs (%)** | 81.8 | 64.5 |  |
| **Methotrexate (%)** | 42.2* | 0.0 |  |
| **Leflunomide (%)** | 15.6* | 0.0 |  |
| **Corticosteroids (%)** | 44.4* | 3.2 |  |
| **Sulfasalazine (%)** | 0.0 | 0.0 |  |
| DAPSA: Disease Activity Score for Psoriatic Arthritis; CRP: C-Reactive Protein; ESR: Erythrocyte Sedimentation Rate; IBD: Inflammatory Bowel Disease; NSAIds: Non-steroidal anti-inflammatory drugs  *Significant differences respect to controls, p-value < 0.05 | | | |

|  | **PsA (n=20)** | **Symptomatic controls (n=20)** |  |
| --- | --- | --- | --- |
| **Female/Male (n/n)** | 12/8 | 11/9 |  |
| **Age (years)** | 50.80 ± 10.76 | 44.53 ± 16.48 |  |
| **Disease duration (years)** | 9.10 ± 6.51 | - |  |
| **Disease activity and inflammatory markers** | | | |
| **DAPSA** | 7.64 ± 7.28 | - |  |
| **CRP (mg/L)** | 2.26 ± 2.82 | 3.23 ± 3.22 |  |
| **ESR (mm/1h)** | 14.06 ± 11.77 | 8.21 ± 7.97 |  |
| **Previous lower back pain (%)** | 15.0* | 95.0 |  |
| **Clinical manifestations/Comorbidities** | | | |
| **Psoriasis (%)** | 75.0* | 15.0 |  |
| **Dactilytis (%)** | 57.9* | 5.0 |  |
| **Uveitis (%)** | 0.0 | 10.0 |  |
| **Enthesitis (%)** | 35.3* | 0.0 |  |
| **Arthritis (%)** | 94.4* | 10.0 |  |
| **IBD (%)** | 6.7 | 20.0 |  |
| **Arterial hypertension (%)** | 20.0 | 15.0 |  |
| **Treatments** | | | |
| **NSAIDs (%)** | 45.0* | 25.0 |  |
| **Methotrexate (%)** | 60.0* | 5.0 |  |
| **Leflunomide (%)** | 20.0* | 0.0 |  |
| **Corticosteroids (%)** | 50.0* | 5.0 |  |
| **Sulfasalazine (%)** | 5.0 | 0.0 |  |
| **Biologics (%)** | 100.0* | 0.0 |  |
| DAPSA: Disease Activity Score for Psoriatic Arthritis; CRP: C-Reactive Protein; ESR: Erythrocyte Sedimentation Rate; IBD: Inflammatory Bowel Disease; NSAIds: Non-steroidal anti-inflammatory drugs  *Significant differences respect to controls, p-value < 0.05 | | | |

**Table S3. Clinical details of validation cohort 2**
